# Risk prediction models for mortality and readmission in patients with acute heart failure: A protocol for systematic review, critical appraisal, and meta-analysis

**DOI:** 10.1371/journal.pone.0283307

**Published:** 2023-07-31

**Authors:** Xuecheng Zhang, Kehua Zhou, Liangzhen You, Jingjing Zhang, Ying Chen, Hengheng Dai, Siqi Wan, Zhiyue Guan, Mingzhi Hu, Jing Kang, Yan Liu, Hongcai Shang

**Affiliations:** 1 Key Laboratory of Chinese Internal Medicine of Ministry of Education, Dongzhimen Hospital, Beijing University of Chinese Medicine, Beijing, China; 2 Beijing University of Chinese Medicine, Beijing, China; 3 Department of Hospital Medicine, ThedaCare Regional Medical Center -Appleton, Appleton, Wisconsin, United States of America; University of Toronto Temerty Faculty of Medicine, CANADA

## Abstract

**Introduction:**

A considerable number of risk models, which predict outcomes in mortality and readmission rates, have been developed for patients with acute heart failure (AHF) to help stratify patients by risk level, improve decision making, and save medical resources. However, some models exist in a clinically useful manner such as risk scores or online calculators, while others are not, providing only limited information that prevents clinicians and patients from using them. The reported performance of some models varied greatly when predicting at multiple time points and being validated in different cohorts, which causes model users uncertainty about the predictive accuracy of these models. The foregoing leads to users facing difficulties in the selection of prediction models, and even sometimes being reluctant to utilize models. Therefore, a systematic review to assess the performance at multiple time points, applicability, and clinical impact of extant prediction models for mortality and readmission in AHF patients is essential. It may facilitate the selection of models for clinical implementation.

**Method and analysis:**

Four databases will be searched from their inception onwards. Multivariable prognostic models for mortality and/or readmission in AHF patients will be eligible for review. Characteristics and the clinical impact of included models will be summarized qualitatively and quantitatively, and models with clinical utility will be compared with those without. Predictive performance measures of included models with an analogous clinical outcome appraised repeatedly, will be compared and synthesized by a meta-analysis. Meta-analysis of validation studies for a common prediction model at the same time point will also be performed. We will also provide an overview of critical appraisal of the risk of bias, applicability, and reporting transparency of included studies using the PROBAST tool and TRIPOD statement.

**Systematic review registration:**

PROSPERO registration number CRD42021256416.

## Introduction

Heart failure is a rapidly growing health issue associated with high mortality and readmission rates [1, 2]. Nearly a third of patients with acute heart failure (AHF) die or are readmitted three months after discharge, accounting for the majority of costs associated with heart failure-related care [3, 4]. Although some improvements have been made in AHF fields, these achievements were realized via adherence to existing chronic heart failure care and improvement in the quality of patient care [5], but not because of new therapeutic developments. In this context, risk stratifications of AHF patients would be useful for more effective, risk-adjusted management [1]. Accurate risk predictions could lead to timely decisions at emergency department (ED) patient triage (discharge with close outpatient follow-up versus hospitalization) and thus the appropriate level of care (outpatient care, ward, telemetry, or intensive care) and its corresponding treatments [6]. Risk prediction models help clinicians stratify patients by their severity of disease or general health condition [7]. Therefore, specialist care and resources could be directed to high-risk patients, while unnecessary testing and procedures could be avoided for low-risk patients, leading to overall healthcare cost savings [8].

Risk prediction models usually encompass a combination of predictors such as vital signs, biomarkers, and demographics; they provide estimates of future outcomes of AHF [9]. A considerable number of multivariable prognostic models have been developed for patients with AHF, and the outcomes most frequently predicted are mortality and readmission rates [7]. Some models shown as a risk score or online calculator [4, 10, 11], making them easily accessible to potential users such as clinicians and patients. Some models were developed as a formula that allows users to assess the risk of death or readmission through complex calculations, although cumbersome [12–14]. However, a certain number of studies reported only limited information about the model [15–17], such as partial predictors or no calculation rules, making it difficult for clinicians and patients to use. Besides, few prediction rules have undergone formal impact analysis to determine whether they improve outcomes when used in clinical practice.

On the other hand, several well-known models had been developed initially for prediction at specific time points and showed good performance [18, 19], but have subsequently been used for validation at other time points and showed significantly different prediction performance at different time points [20, 21]. Even at the same time point, when the same model was validated across the different cohorts, the performance varies considerably [15, 22–26]. It may contribute to uncertainty and confusion among model users about the prediction accuracy of these models.

1. The above problems lead to users facing difficulties in the selection of prediction models, and even sometimes reluctant to utilize models, which limits the application of models [27]. Therefore, a systematic review to assess the performance at multiple time points, applicability, and clinical impact of extant prediction models for mortality and readmission in AHF patients is essential. It may facilitate the selection of models for clinical implementation.

Here we describe our protocol to systematically review the prognostic models for mortality and readmission in patients with AHF. Our specific objectives are: (1) to summarize the characteristics and clinical impact of identified prediction models for mortality and readmission in patients with AHF quantitatively and qualitatively, and compare models with clinical utility with those without, (2) to synthesize the performance of the same model at the same time point when validated in different cohorts, (3) to critically appraise the risk of bias, applicability, and reporting transparency of these studies on prediction models.

## Methods

The protocol is reported with reference to the Preferred Reporting Items for Systematic Reviews and Meta-Analyses Protocols (PRISMA-P) statement [28] (the checklist is shown in the S1 Table) and is conducted using the methodology recommended for systematic review and meta-analysis of prediction models [29]. We will conduct the framing of the review question (Table 1), study design, data extraction, data analysis and critical appraisal under the relevant guidelines [30–32]. We have registered the study on PROSPERO(CRD42021256416).

**Table 1:** Framing of this systematic review using key items identified by the CHARMS checklist.

Items	Comments
1. Prognostic versus diagnostic prediction mode	Aim is to review models predicting future events (prognostic prediction model)
2. Intended scope of the review	Models to inform physicians’ therapeutic decision making
3. Type of prediction modelling studies	Prediction models for acute heart failure patients (derivation and validation), the performance of acute heart failure models (validation), and added value of specific predictor or updating of a specific model (derivation and validation)
4. Target population to whom the prediction model applies	Patients diagnosed as acute heart failure
5. Outcome to be predicted	Mortality and readmission rate
6. Time span of prediction	Within an unlimited time interval (e.g. 7 days, 30 days, 180 days, 1 year or any other specific time interval)
7. Intended moment of using the model	At any time during patients visit to the ED or hospitalization

### Eligibility criteria

Studies will be screened on the basis of the following criteria: population (P), index (I), comparator (C), outcomes (O), timing (T), and setting (S). The PICOTS is an amendment of the PICOS system specific to systematic reviews of prediction models with extra consideration for timing and clinical setting [29, 33].

#### Patients

Studies on prediction models for patients with AHF (or acute decompensated heart failure) will be considered for inclusion, but those that focus exclusively on patients with specific morbidities or groups of children will be excluded. AHF was defined as either new-onset heart failure or decompensation of chronic heart failure.

#### Index/Potential prognostic models

Studies reporting the derivation and validation of a multivariable prediction model will be eligible for this review. The derived model should include at least 50 patients who experienced an event during the period of observation, because studies with fewer cases may not be sufficient for convincing administrative or clinical use [34].

#### Outcome, timing and setting

The primary outcomes predicted by the models under review are mortality and readmission rates of AHF patients. Readmission is defined as all-cause or heart failure-related readmission, which includes emergency department revisits or re-hospitalizations. The studies included in this review should report at least one of the primary outcomes. Studies for prediction models in inpatient and emergency department settings at any period will be included.

#### Types of studies and limits

Cohort, nested case-control, case-cohort, or registry studies using any type of data source (e.g., administrative databases and electronic medical records) will be included in this review. Only those original research articles reported in peer-reviewed publications will be eligible for this review. Secondary research, reviews, conference proceedings, dissertations, editorials, expert opinions, or consensus paper abstracts will be excluded.

### Search strategy

Embase, Pubmed, Web of Science, and the Cochrane Library will be searched for results from their inception onwards to 1 May 2022 to 1 August 2022. Detailed search strategies are presented in S2 Table, and the search terms cover expressions for acute heart failure, prediction model, and mortality/readmission. We will focus on studies published in English, and search a backword citation on all model derivation studies and to identify relevant external validation studies via these studies.

### Study selection

Reviewers will undergo formal training before the formal selection of studies, considering different backgrounds and knowledge of the reviewers. Eligibility criteria will be explained and discussed in detail to ensure the reviewers have the same understanding. Search results will be combined using Endnote X9, and duplicates will be removed. Abstracts and titles of each study will be screened by two reviewers (selected among XZ, SW, ZG, MH), excluding articles based on eligibility criteria.

### Data extraction and management

Two independent reviewers (selected among XZ, SW, ZG, MH) will perform the data extraction, using a standardized data extraction form for all included studies. The form was developed based on the Critical Appraisal and Data Extraction for Systematic Reviews of Prediction Modelling Studies (the CHARMS checklist) [31], and full details of the form are presented in the S3 Table. Disagreements on data element will be resolved via discussion or consultation with a third reviewer when a consensus cannot be reached. We will obtain missing data from the authors if possible. A study will be excluded if the key missing data (e.g., study information, sample size, model performance) could not be supplemented. We will reserve a data records repository for our review which contain relevant information, and it will be available for the public if necessary.

### Critical appraisal

#### Risk of bias and applicability assessment

We will provide an overview critical appraisal of the methodological quality and reporting transparency of included studies. To critically appraise the methodological quality of included studies, which will include the risk of bias and applicability, we will use the Prediction model Risk Of Bias Assessment Tool (PROBAST) [32]. This tool consists of 20 items structured in four domains: participants, predictors, outcome, and analysis. Each domain will be ranked as “high”, “low” or “unclear” for both risk of bias and applicability. Risk of bias contains four domains and applicability contains the first three domains. Additionally, variables number over ten and the inclusion of many continuous variables (>4) will be regarded as barriers to routine use. Detailed items of PROBAST tool applied for our study are in S4 Table.

#### Reporting transparency assessment

The Transparent Reporting of a multivariable prediction model for Individual Prognosis or Diagnosis (TRIPOD statement) [30], which provides a checklist of 22 items considered indispensable for reporting transparency of a prediction model study, will be used for evaluating transparency of the reporting of the included studies. Each element of the TRIPOD statement could be answered with “yes” or “no”, depending on whether the element was reported in the studies. Each “yes” answer will receive 1 point, and each “no” answer will receive 0 points. For elements that do not apply to a specific situation, they can be marked as “not applicable”. We will report the overall TRIPOD adherence score of each study [35], which was developed to assess the uniformity in measuring adherence to the TRIPOD statement. The computational method of TRIPOD adherence score is calculated by dividing the sum of the adhered TRIPOD items by the total number of applicable TRIPOD items. Detailed items of TRIPOD adherence score applied for our study are in S4 Table.

To ensure consistency in evaluating the parameters, reviewers will participate in a pre-study training before the critical appraisal. These parts of the assessment will be conducted by two independent reviewers (XZ and MH). Any disagreement will be handled as described previously.

## Analysis

### Evidence synthesis of qualitative data

Characteristics and clinical impact of the included studies will be described systematically by a narrative synthesis, and quantitative data from the included studies will be presented. Key findings, such as outcomes to be predicted, predictors, performance measures, clinical utility, and the predictive accuracy of the model, will be tabulated to facilitate comparisons. Clinical utility referred to ease of use, defined by whether they exist in a clinically useful manner such as risk score or online calculator. The clinical impact will be measured by the convenience of use, risk of bias, applicability, and accuracy of the model. We will report uncertain measures in the way they were published or approximated using published methods [29].

### Quantitative syntheses

#### Meta-analyses and investigation of heterogeneity

Quantitative synthesis of the predictive performance of the included models will be based on the results of performance measures and their precision values. Derivation and validation of models will be considered separately. Data will be synthesized by a meta-analysis if at least 5 studies are included in a subset with an analogous clinical question appraised repeatedly. A meta-analysis of performance in validation for a common prediction model at the same time point will be performed if at least 3 studies are included.

Performance measures of discrimination (C-statistics, area under the curve) and calibration (O: E ratio, Hosmer-Lemeshow test) will be analyzed by a random-effects model of meta-analysis to estimate the average performance. The C-statistic will be estimated from the reported measure when missing, if necessary [29]. If the data on the total number of expected (E) and observed (O) events are available for extraction among the included models, the O: E ratio will be used for further analysis and roughly provide an indication of the overall model calibration [36]. Extracted C-statistics and total O:E ratios will be rescaled before further meta-analysis to improve the validity of their underlying assumptions if necessary, according to statistic models provided in the literature [37, 38]. To better handle the uncertainty in the estimated heterogeneity among studies, we will adopt the restricted maximum likelihood (REML) estimation and use the Hartung-Knapp-Sidik-Jonkman (HKSJ) method when calculating 95% confidence intervals for the average performance. And the meta-analysis will be performed using Software Stata 16.0 (Stata Corp, College Station, TX, USA) with several packages.

Heterogeneity in the pooled results is usually dependent on differences in the design and populations across the validation studies, such as changes in case mix variation or baseline risk. The case-mix variation of each study will be quantified by estimating the standard deviation of the linear predictor [29]. Cochran’s Q and the I^2^statistic will be calculated for statistical homogeneity assessment of heterogeneity. Potential sources of heterogeneity will be explored using meta-regression analyses if there are enough studies included in the meta-analyses (≥10 studies).

#### Subgroup analysis

If the number of included studies is large enough for each specific subgroups(≥10 studies), we will perform the following subgroup analyses: (1) type of prediction model; (2) method of predictive model building; (3) settings (ED and inpatient); (4) source of data; (5) location, (6) time points. Further subgroup analysis will be dependent on the final data extraction.

#### Sensitivity analysis

Sensitivity analyses will be conducted by excluding studies with a high risk of bias (at least 4/7 domains determined using the PROBAST tool) and studies with low reporting transparency (the overall TRIPOD adherence score <50%) [35], to explore their influence on effect size.

### Reporting and dissemination

We will report our review per guidance by the PRISMA statement [39]. Informed consent and ethical approval are not required because all data will be acquired from published studies. The results of the study will be published in peer-reviewed journals and presented at conferences if possible. Any important protocol modifications will be presented and made available on the PROSPERO registration.

## Discussion

This systematic review protocol will identify existing prognostic models for mortality and readmission in patients with AHF. Characteristics and clinical impact of these prognostic models will be comprehensively summarized, and the reporting transparency and methodological quality of involved studies will be critically appraised. Performance measures of models with an analogous clinical outcome appraised repeatedly and in validation for a common prediction model at the same time point will be synthesized by a meta-analysis.

The result of the planned review would present clearer evidence to clinicians, patients, and researchers, by describing how the confidence in each included study was assessed. Clinicians could reach a better understanding of potential usage scenarios and time points, clinical impact, methodological quality, and limitations of identified models. It may ultimately benefit patients by contributing to more precise, risk-adjusted management, allowing personalized prevention and therapeutic options while decreasing mortality and hospital readmission in AHF. The methodological issues identified by this review may help researchers design more reliable prediction models.

Existing reviews offer limited help for users to judge the performance and clinical impact of models. Meta-analysis for the model performance of the same clinical outcomes and convenience of use is rarely conducted in these reviews, and few relevant reviews have summarized or analyzed the clinical impact, which is the standard of evidence to assess their impact on patients. Without impact analysis, it would be difficult for clinicians to determine whether the use of prediction models is beneficial or harmful [40]. The methodological quality and reporting transparency of the available reviews seem to be absent [6, 9]. Good methodological quality and transparent reporting are essential for clinicians to accurately judge the performance and applicability of prognostic models in real clinical settings of AHF for individualized predictions. Without these criteria, the use of prediction models will be limited due to lack of availability, and sometimes can even be misleading [41]. Besides, a recent review has focused on the models utilized in the ED setting; models exclusively applied to hospitalized patients were excluded from the review [42].

We acknowledge several limitations in our review protocol. First, the included studies may report the model performance using different kinds of measures, and the procedure of merging data may lead to additional bias. Secondly, we will exclude models focusing purely on other outcomes apart from mortality and readmission, such as adverse events [43]. Future studies may extend the scope to all models for other kinds of outcomes and review all AHF prediction models.

## Supporting information

S1 Table PRISMA-P 2015 checklist. (PDF)

S2 Table Search strategies. (PDF)

S3 Table The domains concluded in the data extraction form. (PDF)

S4 Table Detailed items of TRIPOD and PROBAST tool for our study. (PDF)
