# Supplementary material for: Risk prediction models for mortality and readmission in patients with acute heart failure: A protocol for systematic review, critical appraisal, and meta-analysis
Source: PLoS One. 2023 Jul 31;18(7):e0283307. doi: 10.1371/journal.pone.0283307 (PMC10389735; doi:10.1371/journal.pone.0283307)
Supplement: S2 Table — (PDF) [file pone.0283307.s002.pdf]

Supplementary file—appendix 2

Sample search strategies

1. PubMed search strategy

Avalable via https://pubmed.ncbi.nlm.nih.gov

Search date: 1 August 2022

Articles retrieved: 2,484

No.	Searches	Preliminary search
1#	"acute heart failure"[Title/Abstract] OR "acute cardiac failure"[Title/Abstract] OR "acute coronary failure"[Title/Abstract] OR "acute coronary insufficiency"[Title/Abstract] OR "acute decompensated heart failure"[Title/Abstract] OR "acute destabilised heart failure"[Title/Abstract] OR "acute destabilized heart failure"[Title/Abstract] OR "acute heart insufficiency"[Title/Abstract] OR "acutely decompensated heart failure"[Title/Abstract] OR (("acute"[All Fields] OR "acutely"[All Fields] OR "acutes"[All Fields]) AND "destabilised heart failure"[Title/Abstract]) OR "acutely destabilized heart failure"[Title/Abstract] OR "ADHF"[Title/Abstract]	10,326
2#	"risk stratification"[Title/Abstract] OR "prediction model"[Title/Abstract] OR "predict*"[Title/Abstract] OR "progn*"[Title/Abstract] OR "risk prediction"[Title/Abstract] OR "risk score"[Title/Abstract] OR "risk calculation"[Title/Abstract] OR "risk assessment"[Title/Abstract] OR "c statistic"[Title/Abstract] OR "discrimination"[Title/Abstract] OR "calibration"[Title/Abstract] OR "AUC"[Title/Abstract] OR "area under the curve"[Title/Abstract] OR "area under the receiver operator characteristic curve"[Title/Abstract]	2,763,019
3#	"mortality"[Title/Abstract] OR "death"[Title/Abstract]	1,582,745
4#	"readmission"[Title/Abstract] OR "re admission"[Title/Abstract] OR "hospitalization"[Title/Abstract] OR "re-hospitalization"[Title/Abstract]	179,572
5#	#3 OR #4	1,702,106
6#	"English"[Language]	29,631,957
7#	#1 AND #2 AND #5 AND #6	2,484

2. Embase search strategy

Available via https://www.embase.com

Search date: 1 August 2022

Articles retrieved: 1969

No.	Searches	Preliminary search
1#	'acute cardiac failure':ab,ti OR 'acute coronary failure':ab,ti OR 'acute	18941

	coronary insufficiency':ab,ti OR 'acute decompensated heart failure':ab,ti OR 'acute destabilised heart failure':ab,ti OR 'acute destabilized heart failure':ab,ti OR 'acute heart insufficiency':ab,ti OR 'acutely decompensated heart failure':ab,ti OR 'acutely destabilised heart failure':ab,ti OR 'acutely destabilized heart failure':ab,ti OR 'acute heart failure':ab,ti	
2#	'risk stratification':ab,ti OR 'prediction model':ab,ti OR predict*:ab,ti OR progn*:ab,ti OR 'risk prediction':ab,ti OR 'risk score':ab,ti OR 'risk calculation':ab,ti OR 'risk assessment':ab,ti OR 'c statistic':ab,ti OR discrimination:ab,ti OR calibration:ab,ti OR auc:ab,ti OR 'area under the curve':ab,ti OR 'area under the receiver operator characteristic curve':ab,ti	3736724
3#	mortality:ab,ti OR 'death rate':ab,ti OR hospitalization:ab,ti OR rehospitalization:ab,ti OR readmission:ab,ti OR admission:ab,ti	1811551
4#	#1 AND #2 AND #3 AND [humans]/lim AND [clinical study]/lim AND [english]/lim AND [article]/lim	1969
