# Supplementary material for: Risk prediction models for mortality and readmission in patients with acute heart failure: A protocol for systematic review, critical appraisal, and meta-analysis
Source: PLoS One. 2023 Jul 31;18(7):e0283307. doi: 10.1371/journal.pone.0283307 (PMC10389735; doi:10.1371/journal.pone.0283307)
Supplement: S3 Table — (PDF) [file pone.0283307.s003.pdf]

Supplementary file—appendix 3

Data extraction form

Study information	Title, authors, Countries and regions, Published year, Published journal
Type of prediction model	Type of prediction model (subjective prediction, a risk score or regression-based model); development and/or Validation
Source of data	Source of data (e.g., cohort, case-control, randomised trial participants,or registry data)
Participants	Consecutive/inconsecutive participants, location, number of centers, setting(ED,hospitalization,combination,or unclear), AHF/ADHF, age limitation, mean age, Percent of male patients, inclusion and exclusion criteria, study dates
Outcomes to be predicted	Type of outcome(mortality, readmission rate, or rate of death or readmission); time of prediction in days (the period for which the model makes its prediction [e.g., in 1-year mortality, the time of prediction would be 365 days])
Candidate predictors	Number and type of predictors (e.g., demographics, patient history, physical examination, additional testing, disease characteristics), Timing of predictor measurement (e.g., at patient presentation, at diagnosis, at treatment initiation), Handling of predictors in the modelling (e.g., continuous, linear, non-linear transformations or categorised)
Sample size	Total number of participants and number of outcomes/events
Missing data	umber of participants with any missing value, Handling of missing data (e.g., complete-case analysis, imputation, or other methods)
Model development	Modelling method (e.g., logistic, survival, neural networks, or machine learning techniques), Method for selection of predictors for inclusion in multivariable modelling (e.g., all candidate predictors, pre-selection based on unadjusted association with the outcome), Method for selection of predictors during multivariable modelling (e.g., full model approach, backward or forward selection) and criteria used (e.g., p-value, Akaike Information Criterion); The form of statistical analysis used to derive the model (classification and regression tree analysis, Cox proportional hazards regression, generalized linear model or hierarchical modified Poisson regression)

Model performance	Discrimination (C-statistic, D-statistic, log-rank) measures with confidence intervals and calibration (calibration plot, calibration slope, Hosmer-Lemeshow test), classification measures (e.g., sensitivity, specificity, predictive values, net reclassification improvement) and whether a priori cut points were used
Model evaluation	Method used for testing model performance: development dataset only (random split of data, resampling methods, e.g., bootstrap or cross-validation, none) or separate external validation (e.g., temporal, geographical, different setting, different investigators); In case of poor validation, whether model was adjusted or updated (e.g., intercept recalibrated, predictor effects adjusted, or new predictors added)
Results	Final and other multivariable models (e.g., basic, extended, simplified) presented, including predictor weights or regression coefficients, intercept, baseline survival, model performance measures (with standard errors or confidence intervals); Any alternative presentation of the final prediction models, e.g., sum score, nomogram, score chart, predictions for specific risk subgroups with performance ; Comparison of the distribution of predictors (including missing data) for development and validation datasets
Interpretation and Discussion	Interpretation of presented models (confirmatory, i.e., model useful for practice versus exploratory, i.e., more research needed); comparison with other studies, discussion of generalizability, strengths and limitations
