# Supplementary material for: Risk prediction models for mortality and readmission in patients with acute heart failure: A protocol for systematic review, critical appraisal, and meta-analysis
Source: PLoS One. 2023 Jul 31;18(7):e0283307. doi: 10.1371/journal.pone.0283307 (PMC10389735; doi:10.1371/journal.pone.0283307)
Supplement: S4 Table — (PDF) [file pone.0283307.s004.pdf]

PROBAST TOOL

Article Title ______________________________

First author __________ Publication year__________

What type of study is reported?	◯ Development (including internal validation)<br>◯ Development with external validation (same model)<br>◯ Development with external validation (different model)<br>◯ External validation only

Domain 1 : Participants	
Development<br>Were appropriate data sources used, e.g. cohort, RCT or nested case-control study data?	◯ Yes / Probably yes<br>◯ No / Probably no<br>◯ No information
External validation<br>Were appropriate data sources used, e.g. cohort, RCT or nested case-control study data?	◯ Yes / Probably yes<br>◯ No / Probably no<br>◯ No information
Development<br>Were all inclusions and exclusions of participants appropriate?	◯ Yes / Probably yes<br>◯ No / Probably no<br>◯ No information<br>(Yes/probably yes: If inclusion and exclusion of participants was appropriate, so participants correspond to unselected participants of interest. No/probably no: If participants are included who would already have been identified as having the outcome and so are no longer participants at suspicion of disease (diagnostic studies) or at risk of developing outcome (prognostic studies), or if specific subgroups are excluded that may have altered the performance of the prediction model for the intended target population. No information: When there is no information on whether inappropriate inclusions or exclusions took place.)

External validation<br>Were all inclusions and exclusions of participants appropriate?	◯ Yes / Probably yes<br>◯ No / Probably no<br>◯ No information<br>(Yes/probably yes: If inclusion and exclusion of participants was appropriate, so participants correspond to unselected participants of interest. No/probably no: If participants are included who would already have been identified as having the outcome and so are no longer participants at suspicion of disease (diagnostic studies) or at risk of developing outcome (prognostic studies), or if specific subgroups are excluded that may have altered the performance of the prediction model for the intended target population. No information: When there is no information on whether inappropriate inclusions or exclusions took place.)
Development<br>Risk of bias introduced by Participants	◯ Low ROB<br>◯ High ROB<br>◯ Unclear ROB<br>(Low risk of bias: If the answer to all signaling questions is "Yes" or "Probably yes," then risk of bias can be considered low. If ≥1 of the answers is "No" or "Probably no," the judgment could still be "Low risk of bias" but specific reasons should be provided why the risk of bias can be considered low. High risk of bias: If the answer to any of the signaling questions is "No" or "Probably no," there is a potential for bias, except if defined at low risk of bias above. Unclear risk of bias: If relevant information is missing for some of the signaling questions and none of the signaling questions is judged to put this domain at high risk of bias.)
External validation<br>Risk of bias introduced by Participants	◯ Low ROB<br>◯ High ROB<br>◯ Unclear ROB<br>(Low risk of bias: If the answer to all signaling questions is "Yes" or "Probably yes," then risk of bias can be considered low. If ≥1 of the answers is "No" or "Probably no," the judgment could still be "Low risk of bias" but specific reasons should be provided why the risk of bias can be considered low. High risk of bias: If the answer to any of the signaling questions is "No" or "Probably no," there is a potential for bias, except if defined at low risk of bias above. Unclear risk of bias: If relevant information is missing for some of the signaling questions and none of the signaling questions is judged to put this domain at high risk of bias.)
Development<br>Support for Judgement	______________________________
External validation<br>Support for Judgement	______________________________

Domain 2 : Predictors	
Development<br>Were predictors defined and assessed in a similar way for all participants?	◯ Yes / Probably yes<br>◯ No / Probably No<br>◯ No information<br>(Yes/probably yes: If definitions of predictors and their assessment were similar for all participants. No/probably no: If different definitions were used for the same predictor or if predictors requiring subjective interpretation were assessed by differently experienced assessors. No information: If there is no information on how predictors were defined or assessed.)
External validation<br>Were predictors defined and assessed in a similar way for all participants?	◯ Yes / Probably yes<br>◯ No / Probably No<br>◯ No information<br>(Yes/probably yes: If definitions of predictors and their assessment were similar for all participants. No/probably no: If different definitions were used for the same predictor or if predictors requiring subjective interpretation were assessed by differently experienced assessors. No information: If there is no information on how predictors were defined or assessed.)
Development<br>Were predictor assessments made without knowledge of outcome data?	◯ Yes / Probably yes<br>◯ No / Probably no<br>◯ No information
External validation<br>Were predictor assessments made without knowledge of outcome data?	◯ Yes / Probably yes<br>◯ No / Probably no<br>◯ No information
Development<br>Are all predictors available at the time the model is intended to be used?	◯ Yes / Probably Yes<br>◯ No / Probably No<br>◯ No information
External validation<br>Are all predictors available at the time the model is intended to be used?	◯ Yes / Probably Yes<br>◯ No / Probably No<br>◯ No information

Development<br>Risk of bias introduced by predictors	◯ Low ROB<br>◯ High ROB<br>◯ Unclear ROB<br>(Low risk of bias: If the answer to all signaling questions is "Yes" or "Probably Yes," then risk of bias can be considered low. If ≥1 of the answers is "No" or "Probably no," the judgment could still be "Low risk of bias" but specific reasons should be provided why the risk of bias can be considered low, e.g., use of objective predictors not requiring subjective interpretation. High risk of bias: If the answer to any of the signaling questions is "No" or "Probably no," there is a potential for bias. Unclear risk of bias: If relevant information is missing for some of the signaling questions and none of the signaling questions is judged to put the domain at high risk of bias.)
External validation<br>Risk of bias introduced by predictors	◯ Low ROB<br>◯ High ROB<br>◯ Unclear ROB<br>(Low risk of bias: If the answer to all signaling questions is "Yes" or "Probably Yes," then risk of bias can be considered low. If ≥1 of the answers is "No" or "Probably no," the judgment could still be "Low risk of bias" but specific reasons should be provided why the risk of bias can be considered low, e.g., use of objective predictors not requiring subjective interpretation. High risk of bias: If the answer to any of the signaling questions is "No" or "Probably no," there is a potential for bias. Unclear risk of bias: If relevant information is missing for some of the signaling questions and none of the signaling questions is judged to put the domain at high risk of bias.)
Development<br>Support for Judgement	____
External validation<br>Support for Judgement	____
**Domain 3 : Outcome**	
Development<br>Was the outcome determined appropriately?	◯ Yes / Probably yes<br>◯ No / Probably no<br>◯ No information
External validation<br>Was the outcome determined appropriately?	◯ Yes / Probably yes<br>◯ No / Probably no<br>◯ No information
Development<br>Was a pre-specified or standard outcome definition used?	◯ Yes / Probably yes<br>◯ No / Probably no<br>◯ No information

External validation<br>Was a pre-specified or standard outcome definition used?	◯ Yes / Probably yes<br>◯ No / Probably no<br>◯ No information
Development<br>Were predictors excluded from the outcome definition?	◯ Yes / Probably yes<br>◯ No / Probably no<br>◯ No information
External validation<br>Were predictors excluded from the outcome definition?	◯ Yes / Probably yes<br>◯ No / Probably no<br>◯ No information
Development<br>Was the outcome defined and determined in a similar way for all participants?	◯ Yes / Probably yes<br>◯ No / Probably no<br>◯ No information
External validation<br>Was the outcome defined and determined in a similar way for all participants?	◯ Yes / Probably yes<br>◯ No / Probably no<br>◯ No information
Development<br>Was the outcome determined without knowledge of predictor information?	◯ Yes / Probably yes<br>◯ No / Probably no<br>◯ No information
External validation<br>Was the outcome determined without knowledge of predictor information?	◯ Yes / Probably yes<br>◯ No / Probably no<br>◯ No information
Development<br>Was the time interval between predictor assessment and outcome determination appropriate?	◯ Yes / Probably yes<br>◯ No / Probably no<br>◯ No information
External validation<br>Was the time interval between predictor assessment and outcome determination appropriate?	◯ Yes / Probably yes<br>◯ No / Probably no<br>◯ No information
Development<br>Risk of bias introduced by the outcome	◯ Low ROB<br>◯ High ROB<br>◯ Unclear ROB<br>(Low risk of bias: If the answer to all signaling questions is "Yes" or "Probably yes," then risk of bias can be considered low. If ≥1 of the answers is "No" or "Probably no," the judgment could still be low risk of bias, but specific reasons should be provided why the risk of bias can be considered low, e.g., when the outcome was determined with knowledge of predictor information but the outcome assessment did not require much interpretation by the assessor (e.g., death regardless of cause). High risk of bias: If the answer to any of the signaling questions is "No" or "Probably no," there is a potential for bias. Unclear risk of bias: If relevant information about the outcome is missing for some of the signaling questions and none of the signaling questions is judged to put this domain at high risk of bias.)

External validation<br>Risk of bias introduced by the outcome	◯ Low ROB<br>◯ High ROB<br>◯ Unclear ROB<br>(Low risk of bias: If the answer to all signaling questions is "Yes" or "Probably yes," then risk of bias can be considered low. If ≥1 of the answers is "No" or "Probably no," the judgment could still be low risk of bias, but specific reasons should be provided why the risk of bias can be considered low, e.g., when the outcome was determined with knowledge of predictor information but the outcome assessment did not require much interpretation by the assessor (e.g., death regardless of cause). High risk of bias: If the answer to any of the signaling questions is "No" or "Probably no," there is a potential for bias. Unclear risk of bias: If relevant information about the outcome is missing for some of the signaling questions and none of the signaling questions is judged to put this domain at high risk of bias.)
Development<br>Support for Judgement	____________________________________
External validation<br>Support for Judgement	____________________________________

Domain 4 : Analysis

Development<br>Were there a reasonable number of participants with the outcome?	◯ Yes / Probably yes<br>◯ No / Probably no<br>◯ No information<br>(Yes/probably yes: For model development studies, if the number of participants with the outcome relative to the number of candidate predictor parameters is ≥20 (EPV ≥20).* For model validation studies, if the number of participants with the outcome is ≥100. No/probably no: For model development studies, if the number of participants with the outcome relative to the number of candidate predictor parameters is < 10 (EPV < 10).* For model validation studies, if the number of participants with the outcome is < 100. No information: For model development studies, no information on the number of candidate predictor parameters or number of participants with the outcome, such that the EPV cannot be calculated. For model validation studies, no information on the number of participants with the outcome.)

External validation<br>Were there a reasonable number of participants with the outcome?	◯ Yes / Probably yes<br>◯ No / Probably no<br>◯ No information<br>(Yes/probably yes: For model development studies, if the number of participants with the outcome relative to the number of candidate predictor parameters is ≥20 (EPV ≥20).* For model validation studies, if the number of participants with the outcome is ≥100. No/probably no: For model development studies, if the number of participants with the outcome relative to the number of candidate predictor parameters is < 10 (EPV < 10).* For model validation studies, if the number of participants with the outcome is < 100. No information: For model development studies, no information on the number of candidate predictor parameters or number of participants with the outcome, such that the EPV cannot be calculated. For model validation studies, no information on the number of participants with the outcome.)
Development<br>Were continuous and categorical handled appropriately?	◯ Yes / Probably yes<br>◯ No / Probably no<br>◯ No information<br>(Yes/probably yes: If continuous predictors are not converted into ≥2 categories when included in the model (i.e., dichotomized or categorized), or if continuous predictors are examined for nonlinearity using, for example, fractional polynomials or restricted cubic splines, or if categorical predictor groups are defined using a prespecified method. For model validation studies, if continuous predictors are included using the same definitions or transformations, and categorical variables are categorized using the same cut points, as compared with the development study. No/probably no: If categorical predictor group definitions do not use a prespecified method. For model development studies, if continuous predictors are converted into ≥2 categories when included in the model. For model validation studies, if continuous predictors are included using different definitions or transformations, or categorical variables are categorized using different cut points, as compared with the development study. No information: No information on whether continuous predictors are examined for nonlinearity and no information on how categorical predictor groups are defined. For model validation studies, no information on whether the same definitions or transformations and the same cut points are used, as compared with the development study.)

External validation<br>Were continuous and categorical handled appropriately?	◯ Yes / Probably yes<br>◯ No / Probably no<br>◯ No information<br>(Yes/probably yes: If continuous predictors are not converted into ≥2 categories when included in the model (i.e., dichotomized or categorized), or if continuous predictors are examined for nonlinearity using, for example, fractional polynomials or restricted cubic splines, or if categorical predictor groups are defined using a prespecified method. For model validation studies, if continuous predictors are included using the same definitions or transformations, and categorical variables are categorized using the same cut points, as compared with the development study. No/probably no: If categorical predictor group definitions do not use a prespecified method. For model development studies, if continuous predictors are converted into ≥2 categories when included in the model. For model validation studies, if continuous predictors are included using different definitions or transformations, or categorical variables are categorized using different cut points, as compared with the development study. No information: No information on whether continuous predictors are examined for nonlinearity and no information on how categorical predictor groups are defined. For model validation studies, no information on whether the same definitions or transformations and the same cut points are used, as compared with the development study.)
Development<br>Were enrolled participants included in the analysis?	◯ Yes / Probably yes<br>◯ No / Probably no<br>◯ No information
External validation<br>Were enrolled participants included in the analysis?	◯ Yes / Probably yes<br>◯ No / Probably no<br>◯ No information
Development<br>Were participants with missing data handled appropriately?	◯ Yes / Probably yes<br>◯ No / Probably no<br>◯ No information
External validation<br>Were participants with missing data handled appropriately?	◯ Yes / Probably yes<br>◯ No / Probably no<br>◯ No information
Development<br>Was selection of predictors based on univariable analysis avoided?	◯ Yes / Probably yes<br>◯ No / Probably no<br>◯ No information
Development<br>Were complexities in the data (e.g., censoring, competing risks, sampling of control participants) accounted appropriately?	◯ Yes / Probably yes<br>◯ No / Probably no<br>◯ No information

External validation<br>Were complexities in the data (e.g., censoring, competing risks, sampling of control participants) accounted appropriately?	◯ Yes / Probably yes<br>◯ No / Probably no<br>◯ No information
Development<br>Were relevant model performance measures evaluated appropriately?	◯ Yes / Probably yes<br>◯ No / Probably no<br>◯ No information<br>(Yes/probably yes: If both calibration and discrimination are evaluated appropriately (including relevant measures tailored for models predicting survival outcomes). No/probably no: If both calibration and discrimination are not evaluated, or if only goodness-of-fit tests, such as the Hosmer-Lemeshow test, are used to evaluate calibration, or if for models predicting survival outcomes performance measures accounting for censoring are not used, or if classification measures (like sensitivity, specificity, or predictive values) were presented using predicted probability thresholds derived from the data set at hand. No information: Either calibration or discrimination are not reported, or no information is provided as to whether appropriate performance measures for survival outcomes are used (e.g., references to relevant literature or specific mention of methods, such as using Kaplan-Meier estimates), or no information on thresholds for estimating classification measures is given.)
External validation<br>Were relevant model performance measures evaluated appropriately?	◯ Yes / Probably yes<br>◯ No / Probably no<br>◯ No information<br>(Yes/probably yes: If both calibration and discrimination are evaluated appropriately (including relevant measures tailored for models predicting survival outcomes). No/probably no: If both calibration and discrimination are not evaluated, or if only goodness-of-fit tests, such as the Hosmer-Lemeshow test, are used to evaluate calibration, or if for models predicting survival outcomes performance measures accounting for censoring are not used, or if classification measures (like sensitivity, specificity, or predictive values) were presented using predicted probability thresholds derived from the data set at hand. No information: Either calibration or discrimination are not reported, or no information is provided as to whether appropriate performance measures for survival outcomes are used (e.g., references to relevant literature or specific mention of methods, such as using Kaplan-Meier estimates), or no information on thresholds for estimating classification measures is given.)
Development<br>Were model overfitting, under-fitting, and optimism in model performance accounted for?	◯ Yes / Probably yes<br>◯ No / Probably no<br>◯ No information

External validation<br>Were model overfitting, under-fitting, and optimism in model performance accounted for?	◯ Yes / Probably yes<br>◯ No / Probably no<br>◯ No information
Development<br>Do predictors and their assigned weights in the final model correspond to the results from the reported multivariable analysis	◯ Yes / Probably yes<br>◯ No / Probably no<br>◯ No information
External validation<br>Do predictors and their assigned weights in the final model correspond to the results from the reported multivariable analysis	◯ Yes / Probably yes<br>◯ No / Probably no<br>◯ No information
Development<br>Risk of bias introduced by the analysis	◯ Low ROB<br>◯ High ROB<br>◯ Unclear ROB<br>(Low risk of bias: If the answer to all signaling questions is "Yes" or "Probably yes," then risk of bias can be considered low. If ≥1 of the answers is "No" or "Probably no," the judgment could still be low risk of bias, but specific reasons should be provided why the risk of bias can be considered low. High risk of bias: If the answer to any of the signaling questions is "No" or "Probably no," there is a potential for bias. Unclear risk of bias: If relevant information about the analysis is missing for some of the signaling questions but none of the signaling question answers is judged to put the analysis at high risk of bias)
External validation<br>Risk of bias introduced by the analysis	◯ Low ROB<br>◯ High ROB<br>◯ Unclear ROB<br>(Low risk of bias: If the answer to all signaling questions is "Yes" or "Probably yes," then risk of bias can be considered low. If ≥1 of the answers is "No" or "Probably no," the judgment could still be low risk of bias, but specific reasons should be provided why the risk of bias can be considered low. High risk of bias: If the answer to any of the signaling questions is "No" or "Probably no," there is a potential for bias. Unclear risk of bias: If relevant information about the analysis is missing for some of the signaling questions but none of the signaling question answers is judged to put the analysis at high risk of bias)
Development<br>Support for Judgement	______________________________
External validation<br>Support for Judgement	______________________________

Overall assessment of ROB

Development<br>Overall risk of bias	◯ Low risk of bias<br>◯ High risk of bias<br>◯ Unclear risk of bias<br>(Low ROB: If all domains were rated low risk of bias. If a prediction model was developed without any external validation, and it was rated as low risk of bias for all domains, consider downgrading to high risk of bias. Such a model evaluation can only be considered as low risk of bias, if the development was based on a very large data set and included some form of internal validation. High ROB: If ≥1 domain is judged to be at high risk of bias. Unclear ROB: If an unclear risk of bias was noted in ≥1 domain and it was low risk for all other domains.)
External validation<br>Overall risk of bias	◯ Low risk of bias<br>◯ High risk of bias<br>◯ Unclear risk of bias<br>(Low ROB: If all domains were rated low risk of bias. If a prediction model was developed without any external validation, and it was rated as low risk of bias for all domains, consider downgrading to high risk of bias. Such a model evaluation can only be considered as low risk of bias, if the development was based on a very large data set and included some form of internal validation. High ROB: If ≥1 domain is judged to be at high risk of bias. Unclear ROB: If an unclear risk of bias was noted in ≥1 domain and it was low risk for all other domains.)
Any additional comments about ROB on this article?	__________________________________<br>(If there is something in the "PROBAST" that does not fit into the questions of this form - please use this space to detail. Also use this space to detail anything you are unsure about.)

TRIPOD TOOL

TRIPOD adherence score

Publication year

____

First author

____

Title

____

Type of prediction model study

○ development

○ external validation

○ Incremental value

○ development and external validation

Title 1

	Y	N
**i The words developing/development, validation/validating, incremental/added value (or synonyms) are reported in the title**	○	○
**ii The words prediction, risk prediction, prediction model, risk models, prognostic**	○	○
**models, prognostic indices, risk scores (or synonyms) are reported in the title**	○	○
**iii The target population is reported in the title**	○	○
**iv The outcome to be predicted is reported in the title**	○	○

1 Title
D:Score 1 if all elements are scored as “Y”
V:Score 1 if all elements are scored as “Y”
IV:Score 1 if all elements are scored as “Y”
D+V:Score 1 if all elements are scored as “Y”

○ 1

○ 0

2 Abstract
(NA is only for vi)
For D+V，vi=Y if (D2vi=Y AND V2vi(Y OR NA)) OR (D2vi = (Y OR NA) AND V2vi=Y)

	Y	N	NA
**i The objectives are reported in the abstract**	○	○	○
**ii Sources of data are reported in the abstract. E.g. Prospective cohort, registry data, RCT data.**	○	○	○
**iii The setting is reported in the**	○	○	○

	Y	N	NA
**abstract. E.g. Primary care, secondary care, general population, adult care, or paediatric care. The setting should be reported for both the development and validation datasets, if applicable.**			
**iv A general definition of the study participants is reported in the abstract. E.g. patients with suspicion of certain disease, patients with a specific disease, or general eligibility criteria.**	○	○	○
**v The overall sample size is reported in the abstract**	○	○	○
**vi The number of events (or % outcome together with overall sample size) is reported in the abstract. If a continuous outcome was studied, score Not applicable**	○	○	○
**vii Predictors included in the final model are reported in the abstract. For validation studies of well-known models, at least the name/acronym of the validated model is reported. Broad descriptions are sufficient, e.g. 'all information from patient history and physical examination'. Check in the main text whether all predictors of the final model are indeed reported in the abstract.**	○	○	○
**viii The outcome is reported in the abstract**	○	○	○
**ix Statistical methods are described in the abstract. For model development, at least the type of statistical model should be reported. For validation studies a quote like "model's discrimination and calibration was assessed" is considered adequate. If done, methods of updating should be reported.**	○	○	○
**x Results for model discrimination are reported in the abstract. This should be reported separately for development and validation if a study includes both development and validation.**	○	○	○

	Y	N	NA
**xi Results for model calibration are reported in the abstract. This should be reported separately for development and validation if a study includes both development and validation.**	○	○	○
**xii Conclusions are reported in the abstract. In publications addressing both model development and validation, there is no need for separate conclusions for both; one conclusion is sufficient.**	○	○	○

2 Abstract
D:Score 1 if all elements are scored as “Y” or "NA"
V:Score 1 if all elements are scored as “Y” or "NA"
IV:Score 1 if all elements are scored as “Y” or "NA"
D+V:Score 1 if all elements are scored as “Y” or "NA"

○ 1

○ 0

Background and objectives
3a

	Y	N
**i The background and rationale are presented**	○	○
**ii Reference to existing models is included (or stated that there are no existing models)**	○	○

Background and objectives 3a
D:Score 1 if all elements are scored as “Y”
V:Score 1 if all elements are scored as “Y”
IV:Score 1 if all elements are scored as “Y”
D+V:Score 1 if all elements are scored as “Y”

○ 1

○ 0

3b

	Y	N
**It is stated whether the study describes development and/or validation and/or incremental (added) value**	○	○

3b
D:Score 1 if all elements are scored as “Y”
V:Score 1 if all elements are scored as “Y”
IV:Score 1 if all elements are scored as “Y”
D+V:Score 1 if all elements are scored as “Y”

○ 1

○ 0

Source of data 4a

	Y	N
**i The study design/source of data is described. E.g. Prospectively designed, existing cohort, existing RCT, registry/medical records, case control, case series. This needs to be explicitly reported; reference to this information in another article alone is insufficient.**	○	○

4a
D:Score 1 if all elements are scored as “Y”
V:Score 1 if all elements are scored as “Y”
IV:Score 1 if all elements are scored as “Y”
D+V:Score 1 if all elements are scored as “Y”

○ 1

○ 0

4b
(For some elements it may be acceptable if authors in their report specifically reference to another publication (i.e. explicitly mention that the information of that data extraction element is described somewhere else). This is denoted by the answer option R)

	Y	N	R	NA
**i The starting date of accrual is reported**	○	○	○	○
**ii The end date of accrual is reported**	○	○	○	○
**iii The length of follow-up and prediction horizon/time frame are reported, if applicable. E.g. “Patients were followed from baseline for 10 years“ and “10-year prediction of…”; notably for prognostic studies with long term follow-up.If this is not applicable for an article (i.e. diagnostic study or no follow-up), then score Not applicable.**	○	○	○	○

4b
D:Score 1 if all elements are scored as “Y” or “NA” or “R”
V:Score 1 if all elements are scored as “Y” or “NA” or “R”
IV:Score 1 if all elements are scored as “Y” or “NA” or “R”
D+V:Score 1 if all elements are scored as “Y” or “NA” or “R”

○ 1

○ 0

Participants 5a

	Y	N	R
**i The study setting is reported (e.g. primary care, secondary care, general population) E.g.: ‘surgery for endometrial cancer**	○	○	○

	Y	N	R
**patients' is considered to be enough information about the study setting.**			
**ii The number of centres involved is reported.If the number is not reported explicitly, but can be concluded from the name of the centre/centres, or if clearly a single centre study, score Yes.**	○	○	○
**iii The geographical location (at least country) of centres involved is reported. If no geographical location is specified, but the location can be concluded from the name of the centre(s), score Yes.**	○	○	○

5a
D:Score 1 if all elements are scored as "Y" or "R"
V:Score 1 if all elements are scored as "Y" or "R"
IV:Score 1 if all elements are scored as "Y" or "R"
D+V:Score 1 if all elements are scored as "Y" or "R"

○ 1

○ 0

5b

	Y	N
**i In-/exclusion criteria are stated. These should explicitly be stated. Reasons for exclusion only described in a patient flow is not sufficient.**	○	○

5b
D:Score 1 if all elements are scored as "Y"
V:Score 1 if all elements are scored as "Y"
IV:Score 1 if all elements are scored as "Y"
D+V:Score 1 if all elements are scored as "Y"

○ 1

○ 0

5c

	Y	N	NA
**i Details of any treatments received are described.This item is notably for prognostic modelling studies and is about treatment at baseline or during follow-up. The 'if relevant' judgment of treatment requires clinical knowledge and interpretation. If you are certain that treatment was not relevant, e.g. in some diagnostic**	○	○	○

	Y	N	NA
**model studies, score Not applicable.**			

5c
D: Score 1 if element is scored as “Y”; score Not applicable if element isscored as “NA”
V:Score 1 if element is scored as “Y”; score Not applicable if element isscored as “NA”
IV: Score 1 if element is scored as “Y”; score Not applicable if element isscored as “NA”
D+V: Score 1 if element is scored as “Y”; score Not applicable if element is scored as “NA”

○ 1

○ 0

○ NA

Outcome 6a

	Y	N	R
**i The outcome definition is clearly presented. This should be reported separately for development and validation if a publication includes both.**	○	○	○
**ii It is described how outcome was assessed (including all elements of any composite, for example CVD [e.g. MI, HF, stroke]).**	○	○	○
**iii It is described when the outcome was assessed (time point(s) since T0)**	○	○	○

6a
D:Score 1 if all elements are scored as “Y” or “R”
V:Score 1 if all elements are scored as “Y” or “R”
IV:Score 1 if all elements are scored as “Y” or “R”
D+V:Score 1 if all elements are scored as “Y” or “R”

○ 1

○ 0

6b

	Y	N
**i Actions to blind assessment of outcome to be predicted are reported. If it is clearly a non-issue (e.g. all-cause mortality or an outcome not requiring interpretation), score Yes. In all other instances, an explicit mention is expected.**	○	○

6b
D:Score 1 if all elements are scored as “Y”
V:Score 1 if all elements are scored as “Y”
IV:Score 1 if all elements are scored as “Y”
D+V:Score 1 if all elements are scored as “Y”

○ 1

○ 0

Predictors 7a
(Only ii iii iv could be "R")

	Y	N	R
**i All predictors are reported. For development, "all predictors" refers to all predictors that potentially could have been included in the 'final' model (including those considered in any univariable analyses). For validation, "all predictors" means the predictors in the model being evaluated.**	○	○	○
**ii Predictor definitions are clearly presented**	○	○	○
**iii It is clearly described how the predictors were measured**	○	○	○
**iv It is clearly described when the predictors were measured**	○	○	○

7a
D:Score 1 if all elements are scored as "Y" or "R"
V:Score 1 if all elements are scored as "Y" or "R"
IV:Score 1 if all elements are scored as "Y" or "R"
D+V:Score 1 if all elements are scored as "Y" or "R"

○ 1

○ 0

7b

	Y	N
**i It is clearly described whether predictor assessments were blinded for outcome. For predictors for which it is clearly a non-issue (e.g. automatic blood pressure measurement, age, sex) and for instances where the predictors were clearly assessed before outcome assessment, score Yes. For all other predictors an explicit mention is expected.**	○	○
**ii It is clearly described whether predictor assessments were blinded for the otherpredictors**	○	○

7b
D:Score 1 if all elements are scored as "Y"
V:Score 1 if all elements are scored as "Y"
IV:Score 1 if all elements are scored as "Y"
D+V:Score 1 if all elements are scored as "Y"

○ 1

○ 0

8 Sample size

	Y	N
**i It is explained how the study size was arrived at Is there any mention of sample size, e.g. whether this was done on statistical grounds or practical/logistical grounds (e.g. an existing study cohort or data set of a RCT was used)?**	○	○

8
D:Score 1 if all elements are scored as “Y”
V:Score 1 if all elements are scored as “Y”
IV:Score 1 if all elements are scored as “Y”
D+V:Score 1 if all elements are scored as “Y”

○ 1

○ 0

9 Missing data
(i iii iv could be NA)

	Y	N	NA
**i The method for handling missing data (predictors and outcome) is mentioned. E.g. Complete case (explicit mention that individuals with missing values have been excluded), single imputation, multiple imputation, mean/median imputation. If there is no missing data, there should be an explicit mention that there is no missing data for all predictors and outcome. If so, score Yes. If it is unclear whether there is missing data (from e.g. the reported methods or results), score No.If it is clear there is missing data, but the method for handling missing data is unclear, score No.**	○	○	○
**ii If missing data were imputed, details of the software used are given. When under 9i explicit mentioning of no missing data, complete case analysis or no imputation applied, score Not applicable**	○	○	○
**iii If missing data were imputed, a description of which variables were included in the imputation procedure is given. When under 9i explicit mentioning of no missing data, complete case**	○	○	○

	Y	N	NA
**analysis or no imputation applied, score Not applicable**			
**iv If multiple imputation was used, the number of imputations is reported**	○	○	○
**When under 9i explicit mentioning of no missing data, complete case analysis or no imputation applied, score Not applicable**	○	○	○

9
D:Score 1 if all elements are scored as “Y” or "NA"
V:Score 1 if all elements are scored as “Y” or "NA"
IV:Score 1 if all elements are scored as “Y” or "NA"
D+V:Score 1 if all elements are scored as “Y” or "NA"

○ 1

○ 0

Statistical analysis methods 10a

	Y	N	NA
**i For continuous predictors it is described whether they were modelled as linear, nonlinear (type of transformation specified) or categorized. A general statement is sufficient, no need to describe this for each predictor separately. If no continuous predictors were reported, score Not applicable.**	○	○	○
**ii For categorical or categorized predictors, the cut-points were reported. If no categorical or categorized predictors were reported, score Not applicable.**	○	○	○
**iii For categorized predictors the method to choose the cut-points was clearly described. If no categorized predictors, score Not applicable.**	○	○	○

10a
D:Score 1 if all elements are scored as “Y” or "NA
IV:Score 1 if all elements are scored as “Y” or "NA"
D+V:Score 1 if all elements are scored as “Y” or "NA"

○ 1

○ 0

10b
Only ii iii v could be “NA”

	Y	N	NA
**i The type of statistical model is reported. E.g. Logistic, Cox, other regression model (e.g. Weibull, ordinal), other statistical modelling (e.g. neural network)**	○	○	○
**ii The approach used for predictor selection before modelling is described 'Before modelling' means before any univariable or multivariable analysis of predictor-outcome associations. If no predictor selection before modelling is done, score Not applicable. If it is unclear whether predictor selection before modelling is done, score No. If it is clear there was predictor selection before modelling but the method was not described, score No.**	○	○	○
**iii The approach used for predictor selection during modelling is described. E.g. Univariable analysis, stepwise selection, bootstrap, Lasso.'During modelling' includes both univariable or multivariable analysis of predictor-outcome associations.If no predictor selection during modelling is done (so-called full model approach), score Not applicable. If it is unclear whether predictor selection during modelling is done, score No. If it is clear there was predictor selection during modelling but the method was not described, score No.**	○	○	○
**iv Testing of interaction terms is described. If it is explicitly mentioned that interaction terms were not addressed in the prediction model, score Yes. If interaction terms were included in the prediction model, but the testing is not described, score No.**	○	○	○
**v Testing of the proportionality of hazards in survival models is described. If no proportional hazard model is used, score Not applicable.**	○	○	○
**vi Internal validation is reported. E.g.**	○	○	○

	Y	N	NA
**Bootstrapping, cross validation, split sample. If the use of internal validation is clearly a non-issue (e.g. in case of very large data sets), score Yes. For all other situations an explicit mention is expected.**			

10b
D:Score 1 if all elements are scored as “Y” or "NA
IV:Score 1 if all elements are scored as “Y” or "NA"
D+V:Score 1 if all elements are scored as “Y” or "NA"

○ 1

○ 0

10c

	Y	N
**i It is described how predictions for individuals (in the validation set) were obtained from the model being validated. E.g. Using the original reported model coefficients with or without the intercept, and/or using updated or refitted model coefficients, or using a nomogram, spreadsheet or web calculator.**	○	○

10c
D:Score 1 if all elements are scored as “Y”
IV:Score 1 if all elements are scored as “Y”
D+V:Score 1 if all elements are scored as “Y”

○ 1

○ 0

10d

	Y	N
**i Measures for model discrimination are described. E.g. C-index / area under the ROC curve**	○	○
**ii Measures for model calibration are described. E.g. calibration plot, calibration slope or intercept, calibration table, Hosmer Lemeshow test, O/E ratio.**	○	○
**iii Other performance measures are described. E.g. R2, Brier score, predictive values, sensitivity, specificity, AUC difference, decision**	○	○

	Y	N
**curve analysis, net reclassification improvement, integrated discrimination improvement, AIC**		

10d
D:Score 1 if all elements are scored as “Y”
V:Score 1 if all elements are scored as “Y”
IV:Score 1 if all elements are scored as “Y”
D+V:Score 1 if all elements are scored as “Y”

○ 1

○ 0

10e

	Y	N	NA
**i A description of model-updating is given. E.g. Intercept recalibration, regression coefficient recalibration, refitting the whole model, adding a new predictor. If updating was done, it should be clear which updating method was applied to score Yes. If it is not explicitly mentioned that updating was applied in the study, score this item as ‘Not applicable’.**	○	○	○

10e
V:Score 1 if element is scored as “Y”; score Not applicable if element isscored as “NA”
IV: Score 1 if element is scored as “Y”; score Not applicable if element isscored as “NA”
D+V: Score 1 if element is scored as “Y”; score Not applicable if element is scored as “NA”

○ 1

○ 0

○ NA

11 Risk groups

	Y	N	NA
**i If risk groups were created, risk group boundaries (risk thresholds) are specified Score this item separately for development and validation if a study includes both development and validation. If risk groups were not created, score this item as not applicable.**	○	○	○

11
D:Score 1 if all elements are scored as “Y” or "NA"
V:Score 1 if all elements are scored as “Y” or "NA"
IV:Score 1 if all elements are scored as “Y” or "NA"
D+V:Score 1 if all elements are scored as “Y” or "NA"

○ 1

○ 0

12 Development vs. validation
Only IVcould be NA

	Y	N	NA
**i Differences or similarities in definitions with the development study are described. Mentioning of any differences in all four (setting, eligibility criteria, predictors and outcome) is required to score Yes. If it is explicitly mentioned that there were no differences in setting, eligibility criteria, predictors and outcomes, score Yes. For incremental value reports, in case additional predictors are not added to a previously developed prediction model but rather added to conventional predictors in a newly fitted model, score Not applicable.**	○	○	○

12
V:Score 1 if all elements are scored as “Y” or "NA"
IV:Score 1 if all elements are scored as “Y” or "NA"
D+V:Score 1 if all elements are scored as “Y” or "NA"

○ 1

○ 0

Participants 13a
Only ii iii could be NA

	Y	N	NA
**i The flow of participants is reported**	○	○	○
**ii The number of participants with and without the outcome are reported. If outcomes are continuous, score Not applicable.**	○	○	○
**iii A summary of follow-up time is presented. This notably applies to prognosis studies and diagnostic studies with follow-up as diagnostic outcome. If this is not applicable for an article (i.e. diagnostic study or no follow-up), then score Not applicable.**	○	○	○

13a
D:Score 1 if all elements are scored as “Y” or "NA"
V:Score 1 if all elements are scored as “Y” or "NA"
IV:Score 1 if all elements are scored as “Y” or "NA"
D+V:Score 1 if all elements are scored as “Y” or "NA"

○ 1

○ 0

13b

	Y	N
**i Basic demographics are reported**	○	○
**ii Summary information is provided for all predictors included in the final developed/validated model**	○	○
**iii The number of participants with missing data for predictors is reported**	○	○
**iv The number of participants with missing data for the outcome is reported**	○	○

13b
D:Score 1 if all elements are scored as “Y”
V:Score 1 if all elements are scored as “Y”
IV:Score 1 if all elements are scored as “Y”
D+V:Score 1 if all elements are scored as “Y”

○ 1

○ 0

13c
Only IV could be NA

	Y	N	NA
**i Demographic characteristics (at least age and gender) of the validation study participants are reported along with those of the original development study For incremental value reports, in case additional predictors are not added to a previously developed prediction model but rather added to conventional predictors in a newly fitted model, score Not applicable.**	○	○	○
**ii Distributions of predictors in the model of the validation study participants are reported along with those of the original development study. For incremental value reports, in case additional predictors are not added to a previously developed prediction model but rather added to**	○	○	○

	Y	N	NA
**conventional predictors in a newly fitted model, score Not applicable.**			
**iii Outcomes of the validation study participants are reported along with those of the original development study. For incremental value reports, in case additional predictors are not added to a previously developed prediction model but rather added to conventional predictors in a newly fitted model, score Not applicable.**	○	○	○

13c
D:Score 1 if all elements are scored as “Y” or "NA"
V:Score 1 if all elements are scored as “Y” or "NA"
IV:Score 1 if all elements are scored as “Y” or "NA"
D+V:Score 1 if all elements are scored as “Y” or "NA"

○ 1

○ 0

Model development 14a
Only ii could be NA

	Y	N	NA
**i The number of participants in each analysis (e.g. in the analysis of each model if more than one model is developed) is specified**	○	○	○
**ii The number of outcome events in each analysis is specified (e.g. in the analysis of each model if more than one model is developed). If outcomes are continuous, score Not applicable.**	○	○	○

14a
D:Score 1 if all elements are scored as “Y” or "NA"
V:Score 1 if all elements are scored as “Y” or "NA"
IV:Score 1 if all elements are scored as “Y” or "NA"
D+V:Score 1 if all elements are scored as “Y” or "NA"

○ 1

○ 0

14b

	Y	N	NA
**i The unadjusted associations between each**	○	○	○

	Y	N	NA
**predictor and outcome are reported If any univariable analysis is mentioned in the methods but not in the results, score No. If nothing on univariable analysis (in methods or results) is reported, score this item as Not applicable**			

14b
D:Score 1 if element is scored as “Y”; score Not applicable if element is scored as “NA”
IV: Score 1 if element is scored as “Y”; score Not applicable if element is scored as “NA”
D+V: Score 1 if element is scored as “Y”; score Not applicable if element is scored as “NA”

○ 1

○ 0

○ NA

Model specification 15a

	Y	N
**i The regression coefficient (or a derivative such as hazard ratio, odds ratio, risk ratio) for each predictor in the model is reported**	○	○
**ii The intercept or the cumulative baseline hazard (or baseline survival) for at least one time point is reported**	○	○

15a
D:Score 1 if all elements are scored as “Y”
V:Score 1 if all elements are scored as “Y”
IV:Score 1 if all elements are scored as “Y”
D+V:Score 1 if all elements are scored as “Y”

○ 1

○ 0

15b

	Y	N
**i An explanation (e.g. a simplified scoring rule, chart, nomogram of the model, reference to online calculator, or worked example) is provided to explain how to use the model for individualised predictions.**	○	○

15b
D:Score 1 if all elements are scored as “Y”
V:Score 1 if all elements are scored as “Y”
IV:Score 1 if all elements are scored as “Y”
D+V:Score 1 if all elements are scored as “Y”

○ 1

○ 0

16 Model performance

	Y	N	NA
**i A discrimination measure is presented. E.g. C-index / area under the ROC curve**	○	○	○
**ii The confidence interval (or standard error) of the discrimination measure is presented**	○	○	○
**iii Measures for model calibration are described E.g. calibration plot, calibration slope or intercept, calibration table, Hosmer Lemeshow test, O/E ratio.**	○	○	○
**iv Other model performance measures are presented. E.g. R2, Brier score, predictive values, sensitivity, specificity, AUC difference,**	○	○	○
**decision curve analysis, net reclassification improvement, integrated discrimination improvement, AIC.**	○	○	○

16
D: Score 1 if elements 16i- 16iii are scored as "Y"
V: Score 1 if elements 16i- 16iii are scored as "Y"
IV: Score 1 if all elements are scored as "Y"
D+V: Score 1 if elements 16i-16iii are scored as "Y"

○ 1

○ 0

Model updating 17

	Y	N
**i The updated regression coefficients for each predictor in the model are reported. If model updating was described as 'not needed', score Yes.**	○	○
**ii The updated intercept or cumulative baseline hazard or baseline survival (for at least one time point) is reported. If model updating was described as 'not needed', score Yes.**	○	○
**iii The discrimination of the updated model is reported**	○	○
**iv The confidence interval (or standard error) of the**	○	○

	Y	N
**discrimination measure of the updated model is reported**		
**v The calibration of the updated model is reported**	○	○

17
V:Score 1 if all elements are scored as “Y”
D+V:Score 1 if all elements are scored as “Y”

○ 1

○ 0

18 Limitations

	Y	N
**i Limitations of the study are discussed. Stating any limitation is sufficient.**	○	○

18
D:Score 1 if all elements are scored as “Y”
V:Score 1 if all elements are scored as “Y”
IV:Score 1 if all elements are scored as “Y”
D+V:Score 1 if all elements are scored as “Y”

○ 1

○ 0

Interpretation 19a

	Y	N
**i Comparison of results to reported performance in development studies and/or other validation studies is given**	○	○

19a
D:Score 1 if all elements are scored as “Y”
V:Score 1 if all elements are scored as “Y”
IV:Score 1 if all elements are scored as “Y”
D+V:Score 1 if all elements are scored as “Y”

○ 1

○ 0

19b

	Y	N
**i An overall interpretation of the results is given**	○	○

19b
D:Score 1 if all elements are scored as “Y”
V:Score 1 if all elements are scored as “Y”
IV:Score 1 if all elements are scored as “Y”
D+V:Score 1 if all elements are scored as “Y”

○ 1

○ 0

20 Implications

	Y	N
**i The potential clinical use is discussed. E.g. an explicit description of the context in which the prediction model is to be used (e.g. to identify high risk groups to help direct treatment, or to triage patients for referral to subsequent care).**	○	○
**ii Implications for future research are discussed. E.g. a description of what the next stage of investigation of the prediction model should be, such as ” We suggest further external validation”.**	○	○

20
D:Score 1 if all elements are scored as “Y”
V:Score 1 if all elements are scored as “Y”
IV:Score 1 if all elements are scored as “Y”
D+V:Score 1 if all elements are scored as “Y”

○ 1

○ 0

21 Supplementary information

	Y	N
**i Information about supplementary resources is provided**	○	○

21
D:Score 1 if all elements are scored as “Y”
V:Score 1 if all elements are scored as “Y”
IV:Score 1 if all elements are scored as “Y”
D+V:Score 1 if all elements are scored as “Y”

○ 1

○ 0

21 Funding

	Y	N
**i The source of funding is reported or there is explicit mention that there was no external funding involved**	○	○
**ii The role of funders is reported or there is explicit mention that there was no external funding**	○	○

22
D:Score 1 if all elements are scored as “Y”
V:Score 1 if all elements are scored as “Y”
IV:Score 1 if all elements are scored as “Y”
D+V:Score 1 if all elements are scored as “Y”

○ 1

○ 0
